# Cost-effectiveness of adjunctive negative pressure wound therapy in paediatric burn care: evidence from the SONATA in C randomised controlled trial

**DOI:** 10.1038/s41598-021-95893-9

**Published:** 2021-08-17

**Authors:** Cody C. Frear, Bronwyn R. Griffin, Leila Cuttle, Roy M. Kimble, Steven M. McPhail

**Affiliations:** 1 Centre for Children’s Burns and Trauma Research, Level 7, Child Health Research Centre, The University of Queensland, 62 Graham St, South Brisbane, QLD 4101 Australia; 2 Faculty of Medicine, University of Queensland, Herston, Australia; 3 Menzies Health Institute Queensland, Griffith University, Southport, Australia; 4 School of Biomedical Sciences, Faculty of Health, Queensland University of Technology, Brisbane, Australia; 5 Queensland Children’s Hospital, South Brisbane, Australia; 6 Australian Centre for Health Services Innovation and Centre for Healthcare Transformation, School of Public Health and Social Work, Faculty of Health, Queensland University of Technology, Brisbane, Australia; 7 Clinical Informatics Directorate, Metro South Health, Brisbane, Australia

**Keywords:** Paediatric research, Health care economics

## Abstract

Negative pressure wound therapy (NPWT) has been shown to improve clinical outcomes for children with burns by accelerating wound re-epithelialisation. Its effects on healthcare costs, however, remain poorly understood. The aim of this study was to evaluate the cost-effectiveness of NPWT from a healthcare provider perspective using evidence from the SONATA in C randomised controlled trial, in which 101 children with small-area burns were allocated to either standard care (silver-impregnated dressings) or standard care in combination with adjunctive NPWT. The primary outcome, time to re-epithelialisation, was assessed through a blinded photographic review. Resource usage and costs were prospectively recorded for each participant for up to 6 months. Incremental cost-effectiveness ratios and dominance probabilities were estimated and uncertainty quantified using bootstrap resampling. Mean costs per participant—including dressings, labour, medication, scar management, and theatre operations—were lower in the NPWT group (AUD $903.69) relative to the control group (AUD $1669.01). There was an 89% probability that NPWT was dominant, yielding both faster re-epithelialisation and lower overall costs. Findings remained robust to sensitivity analyses employing alternative theatre costs and time-to-re-epithelialisation estimates for grafted patients. In conclusion, adjunctive NPWT is likely to be a cost-effective and dominant treatment for small-area paediatric burns (ANZCTR.org.au:ACTRN12618000256279).

## Introduction

Effective management of paediatric burns poses a common and costly challenge for health systems worldwide. The fifth leading cause of non-fatal childhood trauma, burns affecting a total body surface area (TBSA) of under 20% often require care from specialised centres staffed with multidisciplinary personnel to ensure adequate analgesia, wound coverage, infection prevention, and scar minimisation^1^. Small-area injuries that make up the majority of paediatric cases are now typically treated in an outpatient setting thanks to silver-impregnated dressings such as Acticoat™ (Smith & Nephew, Hull, UK) and Mepilex Ag™ (Mölnlycke Healthcare, Mikkeli, Finland), which, unlike other common therapies (e.g., silver sulphadiazine), do not require daily dressing changes^2–4^.

Despite advances in wound care, these injuries still carry the risk of serious morbidity. Hypertrophic scarring remains a prevalent post-burn complication, observed in 16–35% of paediatric patients^5–9^. One of the foremost predictors of hypertrophic scar formation is time to re-epithelialisation^6,9, 10^, with long-term consequences and financial costs for patients, their families, and health systems if healing is delayed^11–13^.

Negative pressure wound therapy (NPWT) has been proposed as a substitute or adjunctive treatment that might improve healing outcomes in partial-thickness burns^14,15^. The intervention generates a subatmospheric pressure across a wound surface, which has been found in multiple settings to improve perfusion, reduce oedema, maintain a moist environment, and stimulate angiogenesis and granulation tissue synthesis^16,17^. High-quality evidence of the therapy’s efficacy in acute burn care is scarce but growing^18–22^.

Given the high existing costs of burn management^23^, an essential consideration in the decision surrounding whether to integrate NPWT into standard practice is its cost-effectiveness. Previous studies have found it to be cost-effective in some settings (such as caesarean section incisions in obese women^24,25^, diabetic foot ulcers^26^, split-skin grafts for lower limb skin cancer^27^, severe chronic wounds with multiple comorbidities^28^, and pressure ulcers^29^), but not others (including open fractures and closed surgical wounds of the lower limb^30,31^). This study was performed to investigate the cost-effectiveness of adjunctive NPWT in the treatment of small-area burns in children, using evidence from the Study Of Negative pressure wound therapy as an Adjunctive Treatment for Acute burns in Children (SONATA in C) randomised controlled trial^32,33^.

## Results

A total of 101 children with partial-thickness thermal burns were enrolled and analysed in the SONATA in C trial, of whom 54 were randomised to the control group and 47 to the NPWT group. Full details on the trial’s patient demographics, baseline characteristics, and clinical outcomes are reported elsewhere^33^. In summary, the median patient age was 4 years (IQR 1–8.5), 59 (58.4%) participants were male, the median TBSA was 1.5% (IQR 1–2), and 68 (67.3%) injuries were superficial partial-thickness. Median time to re-epithelialisation was 8 (IQR 7–11) days in the NPWT group and 10 (IQR 8–14) days in the control group. The primary clinical analysis adjusting for depth, anatomical location, and aetiology showed that NPWT decreased the expected time to re-epithelialisation by 22% (95% CI 7–34%; *P* = 0.005). Relevant clinical outcomes are described briefly in Table 1.

**Table 1:** Clinical outcomes for each treatment group.

	Control	NPWT
	(n = 54)	(n = 47)
Time to re-epithelialisation (days)^a^	10 (8–14)	8 (7–11)
Split-thickness skin grafts	4 (7.0%)	1 (2.1%)
Dressing changes under general anaesthetic^b^	3 (5.3%)	1 (2.1%)
Scar management referrals	15 (26.3%)	5 (10.6%)

Values are n (%) unless indicated otherwise.

^a^Median (IQR).

^b^Undertaken in participants at either their second or third clinical visit.

### Costs

The costs accrued by each treatment group, and their breakdown by category, are presented in Table 2 and Supplementary Table S1. The mean total cost for the control group was $1669.01 (95% CI $659.06–$3269.16), compared to $903.69 (95% CI $670.68–$1234.74) for the NPWT group. In the control group, four participants underwent a total of seven operations. In the NPWT group, two participants were each taken to theatre once. Supplementary Table S2 details theatre cost estimates.

**Table 2:** Clinical costs for each treatment group.

Group mean (95% CI^a^) in AUD$	Control	NPWT
Dressing costs	260.16 (186.47–354.19)	441.86 (380.15–521.00)
Analgesia costs	6.39 (4.30–10.52)	4.49 (3.82–5.33)
Acute labour costs	276.79 (236.69–325.01)	268.22 (227.11–316.91)
Scar management costs	31.54 (13.34–57.24)	16.11 (4.37–34.51)
Surgical costs^b^	1094.13 (100.40–2673.49)	173.01 (0.00–464.10)
Total costs	1669.01 (659.06–3269.16)	903.69 (670.68–1234.74)

^a^95% CI derived from bootstrap resampling (2000 replications) to represent distribution uncertainty.

^b^Including grafting and dressing changes under general anaesthetic for patients who underwent a theatre operation.

### Incremental cost-effectiveness

**Figure 1:**
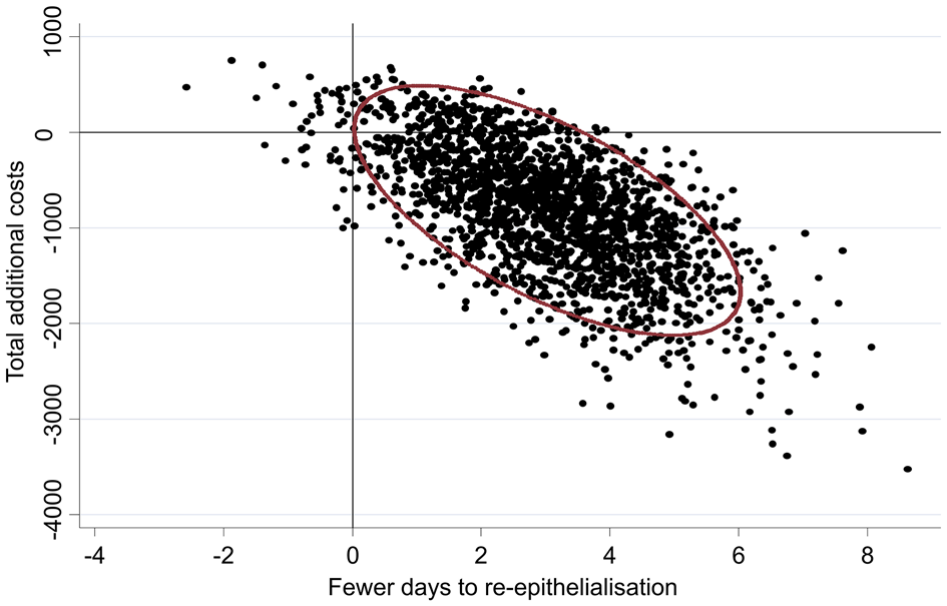
Incremental cost-effectiveness of adjunctive NPWT versus standard care with 95% confidence ellipse.

The primary incremental cost-effectiveness ratio (ICER) estimates are displayed in Fig. 1. Based on bootstrap resampling of costs and effects, there was a 98% probability that time to re-epithelialisation was shorter in the NPWT group, with a mean reduction per participant of 3.19 (95% CI 0.43–5.95) days. The probability that the NPWT group accrued lower overall costs per participant was 90%. Mean cost savings per participant totalled $948.57 (95% CI − $312.03 to $2209.17). There was an 89% probability that NPWT was dominant (that is, yielded faster re-epithelialisation and lower overall costs than standard care).

### Sensitivity analyses

**Figure 2:**
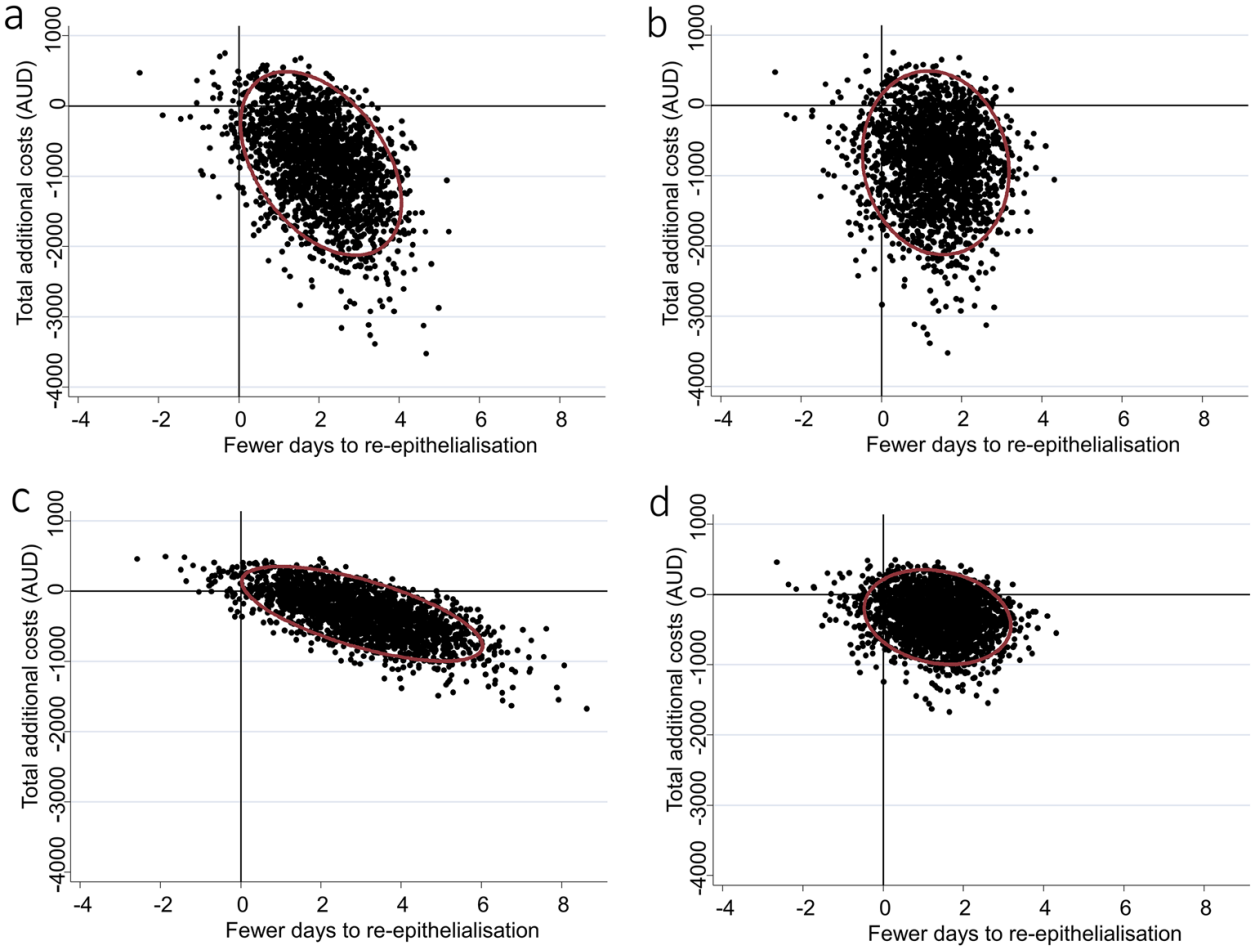
Incremental cost-effectiveness (and 95% confidence ellipses) from the sensitivity analyses using (**a**) an alternative artificial period of 24 days as an estimate of time to re-epithelialisation for grafted patients, (**b**) time until surgery as a proxy for time to re-epithelialisation for grafted patients, (**c**) theatre cost estimates reduced by 50%, and (**d**) a combined approach employing both time until surgery as a proxy for time to re-epithelialisation for grafted patients and theatre cost estimates reduced by 50%.

The above primary analysis imputed an artificial time to re-epithelialisation for grafted patients of 41 days. For the first sensitivity analysis, a more conservative alternative artificial time of 24 days for grafted patients was utilised. This sensitivity analysis also yielded a 98% probability that re-epithelialisation was faster in the NPWT group. The mean decrease in time to re-epithelialisation per participant was 2.12 (95% CI − 0.29 to 3.94) days, and the probability that NPWT dominated standard care was 88% (Fig. 2a). In the second, most extreme conservative sensitivity analysis for time to re-epithelialisation, time to grafting was used for patients who required skin grafts. This analysis indicated a 92% probability of faster re-epithelialisation in the NPWT group, with a mean reduction per participant of 1.36 (95% CI − 0.29 to 3.02) days. There was an 83% probability that NPWT dominated standard care (Fig. 2b).

Given the lack of patient-specific theatre costings and the high cost estimates for skin grafts and dressing changes under general anaesthetic undertaken in a small number of patients, a third sensitivity analysis was conducted in which per-operation theatre cost estimates were halved. With this more conservative approach to costing, there was an 82% probability of lower overall costs per participant in the NPWT group, with mean cost savings per participant of $387.13 (95% CI − $262.91 to $1037.17). There was an 82% probability that NPWT dominated standard care (Fig. 2c).

The final sensitivity analysis, combining both a 50% discount to theatre costs and the most extreme conservative imputation of time to surgery as time to re-epithelialisation for grafted cases, the probability that NPWT dominated standard care remained high at 77% (Fig. 2d).

## Discussion

This study presents the first economic evaluation of adjunctive NPWT in the acute management of small-area thermal burns in children. Results from the SONATA in C randomised controlled trial showed a high probability that NPWT dominated standard care (Acticoat™ with Mepitel™), yielding both faster re-epithelialisation and lower overall healthcare costs. This finding remained robust to sensitivity analyses using alternative time to re-epithelialisation estimates for grafted patients and more conservative theatre cost estimates.

Prior to this trial, there had been limited evidence that NPWT might facilitate physiological healing in burn wounds^14,22^. Its mechanism of action has yet to be characterised definitively, but it is likely multifactorial, with research indicating potential activity in reducing oedema formation and burn wound conversion, modulating inflammation and extracellular matrix degradation, and stimulating neovascularisation and cellular proliferation^16,17,34, 35^. However, the therapy’s effects on overall healthcare costs in acute burn management have thus far remained largely unexplored^18,19^, notwithstanding some limited investigations into its cost-effectiveness as a bolster for skin grafts^36,37^. None of the previously-published randomised controlled trials focusing on spontaneous healing with NPWT reported the collection of any cost data^20–22^.

The present study’s finding that the intervention increased per-participant dressing costs was not unexpected, given that the NPWT system incorporated the control treatment—Acticoat™ with Mepitel™—into an apparatus also composed of adhesive, gauze, soft ports, tubing, and a vacuum pump. Acute non-surgical labour costs were similar across the two treatment groups, even though NPWT required additional time for application and removal. In many cases, management of the intervention also necessitated the involvement of more clinicians than was typical with standard dressings. Children in the NPWT group, however, required fewer dressing changes until re-epithelialisation, which likely offset these additions to overall labour. It is also worth noting that, in contrast with prior trials reporting that NPWT decreased labour costs by reducing hospital length of stay and the frequency of dressing procedures^38,39^, the present study took place predominantly in an outpatient setting with an interval of 3–5 days between changes of dressings for all participants.

The cost categories in which NPWT yielded the greatest reductions were theatre operations and, to a lesser degree, scar management. Participants were referred to scar management if re-epithelialisation exceeded 14 days, grafting was undertaken, or clinicians otherwise deemed their wound to be at high risk of functionally or cosmetically detrimental scarring. Children in the NPWT group showed a significant decrease in the risk of referral to scar management, which was likely attributable to faster re-epithelialisation^33^. This corresponded to a decline in per-participant costs of occupational therapy labour and scar products such as pressure garments and silicone gel. Although the total number of theatre interventions, nine, was too small to allow for inferential statistical analyses, it is noteworthy that seven of these involved participants in the control group, including four of the five skin grafts. Due to their high demands in terms of labour, equipment, and theatre usage, these interventions are particularly costly. The estimated costs of a high-complexity skin graft, derived from patient-level hospital costings, totalled $13,576.70, which was in line with previous reporting in a similar patient population^40^. The cost-related sensitivity analyses, which applied a 50% discount to surgical cost estimates, produced a similar pattern of findings to the primary analysis albeit with a smaller magnitude of per-person cost savings.

Several strengths and limitations should be considered when interpreting these results. One notable asset was the study’s prospective data collection alongside a randomised clinical trial, from which robust effect estimates were generated. On the other hand, the economic evaluation was limited to the healthcare provider perspective, focusing solely on costs for burn-related healthcare in a 6-month time horizon, consistent with the trial follow-up period^32,33^. Productivity losses, informal care, transportation, counselling, and home modifications, while important, were all outside the scope of this research. Similarly, any scar therapies (e.g., surgical reconstructions, microneedling, and laser treatments) that might have been provided beyond the trial follow-up period were not captured. Most scar regimens, however, are completed within the 6-month window used in the present study, which generally encompasses the peak in physical scar severity^41, 42^.

This economic evaluation was conducted in a single-site metropolitan specialist paediatric hospital, and results therefore may not be applicable to dissimilar services and jurisdictions. Nevertheless, treatments were in line with evidence-based best practice, and the standard dressings provided to all participants are widely employed and recommended for paediatric burn care^2–4,43^. The NPWT pump utilised in the trial, the RENASYS TOUCH™, is one of many such devices currently available^44^. Although the investigators consider it to be representative of most portable devices used in the outpatient setting, the study’s findings should not be assumed to generalise to all NPWT systems. Further research is warranted, particularly to examine the cost-effectiveness of ultraportable systems^45,46^, for which dressing and labour costs may be substantially lower.

In conclusion, this trial-based economic evaluation indicated that adjunctive NPWT was likely to be a dominant strategy in the acute management of < 5% TBSA thermal burns in children. Relative to standard care with Acticoat™ and Mepitel™ alone, the intervention was likely to have both hastened re-epithelialisation and reduced overall burn-related healthcare costs. The study provides clinical and economic support for the use of NPWT in the treatment of patients with similar characteristics to those in this trial, although there is a need for larger, multi-centre investigations to validate these findings.

## Methods

### Trial background

The reporting of this economic evaluation conforms to the Consolidated Health Economic Evaluation Reporting Standards (CHEERS)^47^. The SONATA in C trial was a pragmatic, single-centre, two-parallel-arm, randomised, active-controlled trial conducted at a tertiary children’s hospital in Australia between May 2018 and June 2019. Eligible patients included children under 17 years of age who presented with a < 5% TBSA thermal burn no more than 7 days following injury. Facial burns and wounds suspected by clinicians to achieve full re-epithelialisation with standard care by their next appointment 3–5 days later were excluded. Participants were randomised on a 1:1 basis to standard dressings or a combination of standard dressings and NPWT. The trial was registered prior to the start of recruitment with the Australian New Zealand Trial Registry on 16/02/2018 (Registration ID: ACTRN12618000256279) and had the approval of the University of Queensland Human Research Ethics Committee (2018000335/HREC/17/QRCH/279) and the Children’s Health Queensland Hospital and Health Service Human Research Ethics Committee (HREC/17/QRCH/279, SSA/17/QRCH/292). It was performed in accordance with the principles of the Declaration of Helsinki. Written informed consent was obtained from the legal guardians of all participants before study enrolment.

### Design

This economic evaluation took the form of an incremental cost-effectiveness analysis nested in a randomised controlled trial. It was conducted from a healthcare provider perspective to inform clinicians’ decision-making regarding the implementation of NPWT as an adjunctive therapy to reduce time to re-epithelialisation. Cost-effectiveness was evaluated over a 6-month time horizon, coinciding with the follow-up period for the SONATA in C trial^32,33^. Discounting for costs or effects was therefore not warranted.

### Intervention

The control group received the standard-of-care dressings in the participating centre: the nanocrystalline silver-impregnated fibre mesh Acticoat™, with the silicone layer Mepitel™ (Mölnlycke Healthcare, Mikkeli, Finland) serving as a wound interface. In the NPWT group, these dressings were integrated into a NPWT apparatus involving a RENASYS TOUCH™ (Smith & Nephew, Hull, UK) vacuum pump, which delivered a continuous subatmospheric pressure of 80 mmHg (or 40 mmHg, in children under 12 months of age with extremity burns). Dressing changes were conducted every 3–5 days until participants were discharged from the centre or referred to scar management.

### Intervention effect

Time to re-epithelialisation was assessed by means of a blinded review of clinical images. Standard photographic documentation was performed at every change of dressings throughout participants’ acute management. De-identified photographs were judged by a blinded panel of three experienced burn clinicians, who were asked to identify the point at which wounds were ≥ 95% re-epithelialised. Times favoured by at least two of the judges were included in the statistical analysis. In two cases where there was no agreement, a fourth burn clinician intervened to assist in reaching a consensus.

For participants who underwent skin grafting and for whom spontaneous wound closure was not directly observable, time to re-epithelialisation was imputed as 1 day greater than the longest time recorded in the trial for a patient to re-epithelialise without grafting (40 days). This approach was consistent with the primary clinical analysis from this trial and previous research in the field^33,40^.

### Resource usage and costs

Following a bottom-up, micro-costing approach, costs were calculated by prospectively recording the materials, equipment, and personnel directly involved in the care of each participant’s burn wound. Documentation of participants’ clinical visits covered their initial presentation to the participating hospital, all subsequent dressing changes, and any theatre operations and scar management appointments up to 6 months post-re-epithelialisation. During the acute management phase, nursing staff time per dressing application/change was recorded, encompassing the length of removal of previous dressings (if any), cleaning of the wound, administration of new dressings, and an additional three minutes for administrative tasks and documentation (based on an average recorded by the nursing staff). For other personnel, standard per-appointment clinic consultation times were applied as follows: surgical residents, registrars, and consultants (15 min); physiotherapists (15 min); social workers (30 min); and occupational therapists (30 min for dressing change appointments and 45 min for scar management visits). Labour resource usage was costed according to the relevant 2018 state-based industrial award rates for nurses^48^, doctors^49^, and other health practitioners^50^, with an additional 25.85% to account for on-costs including leave accrual and superannuation.

The size and quantity of the applied Acticoat™ and Mepitel™ were recorded, along with any supplementary materials (e.g., adhesive, tubular support bandages, absorbent pads) used to secure the dressings, control oedema, and reduce trauma to the wound. The NPWT apparatus consisted of the gauze packing material, adhesive film, soft port and attached tubing, canister, and pump. For the latter, costs were calculated by applying a per-day cost supplied by the hospital (covering use of both the device itself and a charging pack) to the period of application. Doses of pharmacological analgesia were calculated by weight. Per the clinic’s standard practice, patients received oxycodone (0.1–0.2 mg/kg), acetaminophen (15 mg/kg), and/or ibuprofen (10 mg/kg), with Entonox™ (nitrous oxide/oxygen; BOC Healthcare, Manchester, UK) employed at the discretion of the treating clinicians. At scar management appointments, the type, size, and quantity of all silicone products and pressure garments prescribed by the occupational therapists were recorded. Dressings, medications, and scar management products were costed according to the market prices obtained from the participating hospital’s consumables pricing list. Presentation of unit costs was prohibited by hospital policy.

The costs of theatre operations were estimated using patient-level clinical costings obtained from the participating hospital’s costing department for operations allocated burn-specific Diagnosis-Related Group codes in the 2018–2019 financial year. Because theatre operations ranged widely in cost, operations were divided into low- and high-complexity cases based on their duration (i.e., whether total theatre time exceeded 60 min) and the involvement of sensitive anatomical areas such as the fingers and toes. Prior auditing of clinical costings for theatre cases indicated that these two factors were associated with substantially higher theatre costs. Mean costs in each complexity group were then calculated for split-thickness skin grafts and changes of dressing under general anaesthetic, and applied to the relevant theatre operations involving trial participants. Costs were applied in Australian dollars using 2018 as the base year.

### Incremental cost-effectiveness

An incremental cost-effectiveness analysis was conducted to investigate the cost per one less day to re-epithelialisation. ICER estimates were calculated using the following formula:

$$ICER=\frac{\left(Cost \,NPWT \,group\right)-(Cost \,control \,group)}{\left(Effect \,NPWT \,group\right)-(Effect \,control \,group)}$$

The difference in days to re-epithelialisation was estimated using a multiple regression model adjusted for wound depth, anatomical location, and aetiology, in line with the primary analysis of clinical effect from this trial^33^. Multiple regression adjusting for the same covariates was also employed to estimate the difference in cost. To account for potential uncertainty, bootstrap resampling was undertaken, replicating the original sample 2000 times. The bootstrap resampling was used to derive 95% confidence intervals for differences in cost and time to re-epithelialisation, as well as 95% confidence ellipses plotted on a cost-effectiveness plane consistent with prior research in the field^40^. The proportion of resampled estimates located in the southeast quadrant of the cost-effectiveness plane were used to estimate the probability that NPWT was dominant.

### Sensitivity analyses

To examine whether findings were robust to alternative approaches for estimating time to re-epithelialisation and costs for patients who required theatre operations, four sensitivity analyses were conducted. The first repeated the primary analysis, but for patients who required skin grafting, a period of 24 days was used as a proxy for time to re-epithelialisation. This estimate was based on a hypothetical timeline in which the decision to operate is made at day 14 post-burn, surgery is conducted at day 17, and final wound healing occurs 7 days post-operatively. The second sensitivity analysis employed the time elapsed from injury to the day of surgical grafting as the most extreme conservative estimate of time to re-epithelialisation for participants who received grafting. Seeking to examine whether findings were sensitive to any inadvertent inaccuracies in theatre cost estimates, the third sensitivity analysis repeated the primary analysis applying a 50% discount to all theatre operations (i.e., skin grafts and dressing changes under general anaesthetic). A two-way sensitivity analysis combined the latter two most conservative alternative estimates (i.e., time to surgery and theatre costs reduced by 50%) was utilised in the fourth and final sensitivity analysis. Statistics were carried out using Stata^®^ version 16 (StataCorp LLC College Station, TX, USA).

## Supplementary Information

Supplementary Information 1.

## Data Availability

The datasets used and/or analysed during the current study are available from the corresponding author on reasonable request. Release of data must first be approved by the Children’s Health Queensland Human Research Ethics Committee.
