# Supplementary material for: Cost-effectiveness of adjunctive negative pressure wound therapy in paediatric burn care: evidence from the SONATA in C randomised controlled trial
Source: Sci Rep. 2021 Aug 17;11:16650. doi: 10.1038/s41598-021-95893-9 (PMC8371025; doi:10.1038/s41598-021-95893-9)
Supplement: Supplementary file 1 — Supplementary Information 1. [file 41598_2021_95893_MOESM1_ESM.docx]

**Supplementary Table S1**. Median (IQR) clinical costs for each treatment group

| Group median (IQR) in AUD$ | Control | NPWT |
| --- | --- | --- |
| Dressing costs | 138.78 (90.93 to 254.41) | 351.54 (285.31 to 571.30) |
| Analgesia costs | 3.91 (2.59 to 6.73) | 3.61 (2.72 to 5.96) |
| Acute labour costs | 217.56 (152.61 to 364.52) | 222.42 (161.76 to 312.85) |
| Scar management costs | 0.00 (0.00) | 0.00 (0.00) |
| Surgical costs* | 0.00 (0.00) | 0.00 (0.00) |
| Total costs | **351.36 (265.41-889.65)** | **616.68 (464.85-890.51)** |

*Including grafting and dressing changes under general anaesthetic for patients who underwent a theatre operation

**Supplementary Table S2**. Theatre cost estimates

| Theatre operation | Estimated cost  (AUD$) | Control  (n) | NPWT  (n) |
| --- | --- | --- | --- |
| High-complexity skin graft | $13,576.70 | 2 | 0 |
| Low-complexity skin graft | $4,116.47 | 2 | 1 |
| High-complexity dressing change under general anaesthetic | $9,840.90 | 2 | 0 |
| Low-complexity dressing change under general anaesthetic | $4,015.02 | 1 | 1 |
